# Editorial: Improved diagnostic microbiology for efficient AMR surveillance

**DOI:** 10.3389/fcimb.2026.1989367

**Published:** 2026-10-12

**Authors:** Saffiatou Darboe, Abdoulie Bojang, Luria Leslie Founou, Ibrahim Bitar

**Affiliations:** 1 Partners In Health, Boston, MA, United States; 2 National Institutes of Health, Bethesda, MD, United States; 3 Reproductive, Maternal, Newborn and Child Health (ReMARCH) Research Unit, Research Institute of the Centre of Expertise and Biological Diagnostic of Cameroon (CEDBCAM-RI);, Yaounde, Cameroon; 4 Antimicrobial Research Unit, School of Health Sciences, College of Health Sciences, University of KwaZulu-, Natal, Durban, South Africa; 5 Infection & Global Health Division, School of Medicine, University of St Andrews, St Andrews, United Kingdom; 6 Charles University, Pilsen/Prague, Czechia

**Keywords:** antimicrobial resistance, genomics, low- and middle-income countries, rapid diagnostic, whole genome sequencing

Antimicrobial resistance (AMR) was directly responsible for an estimated 1.27 million deaths in 2019 and associated with nearly 5 million more, with the heaviest burden falling on low- and middle-income countries (LMICs) (Murray et al., 2022). The global response to this burden, through the World Health Organisation (WHO) Global Antimicrobial Resistance and Use Surveillance System (GLASS) (Glass, 2017; WHO, 2020), national action plans, and priority pathogen lists (WHO, 2024), rests on an assumption that is rarely examined: that resistant organisms are being documented before empirical therapy commits the clinical decision. An isolate that is never sought before the first dose is unlikely to be recovered afterwards and never enters any surveillance database. It is precisely this dependency that the Montreal Declaration on AMR addressed when it called for secure budgetary support for routine diagnostic bacteriology, and it is the reason quality-assured laboratory capacity remains the rate-limiting step for AMR containment in most LMIC settings (Ombelet et al., 2018).

This Research Topic invited contributions on how improved diagnostic microbiology, meaning quality improvement, molecular assays, and the integration of genomics into routine laboratories, adds value to AMR surveillance and outbreak investigation. The five articles collected here, from 37 authors, can be read as a sequence along the diagnostic pathway: whether a pathogen test is ordered before antimicrobial therapy begins, what routine results reveal when aggregated over time, how rapid genotypic testing extends resistance-guided therapy beyond the culture laboratory, how culture-independent sequencing performs against phenotypic testing, and what genome-level resolution contributes to transmission investigation.

## The weakest link: testing before treatment

Huang et al. begin at the start of the pathway, analyzing 534,374 emergency department visits across two campuses of a Chinese tertiary hospital between 2021 and 2024. Antimicrobial use rose from 17.95% to 22.59% of visits over the period, while the pre-treatment pathogen testing rate remained flat at 13-14%, producing what the authors describe as a scissor gap between high prescribing and low testing. The composition of what was submitted before therapy is equally telling: procalcitonin dominated throughout, testing for *Mycoplasma pneumoniae* surged after 2023, and conventional bacterial cultures never exceeded 5% of pre-treatment specimens, falling below 1% of antibiotic-treated visits when calculated per encounter. Substantial variation between campuses and between medical and surgical teams pointed to workflow and governance rather than case-mix as the drivers.

The implication for surveillance is uncomfortable. Where a culture is obtained before treatment in fewer than one in a hundred treated patients, resistance rates derived from routine data describe a small and non-random subset of infections, typically the sickest or the treatment failures, and both numerator and denominator are distorted (Li et al., 2025). Specimens taken after therapy has begun do not close the gap, since prior antimicrobial exposure suppresses recovery and biases the organisms that do grow. The authors’ proposed remedies are pragmatic and transferable: multidisciplinary collaborative governance model, electronic prompts tied to restricted-agent prescribing, simplified submission workflows, long term supervision and incentive mechanism, and a data-driven evaluation framework embedding process and outcome indicators.

## What routine data reveal over a decade

Yang et al. show what accumulates when routine microbiology is systematically curated. Their ten-year cohort of 2,669 patients with culture-confirmed hospital-acquired infections spans China’s pre-pandemic, strict containment, and post-adjustment policy phases. Overall multidrug-resistant organism detection rose from 36.9% to 56.4% during strict containment; carbapenem-resistant Enterobacterales displaced carbapenem-resistant *Acinetobacter baumannii* as the dominant phenotype, with *Klebsiella pneumoniae* reaching 40% of isolates in 2022, and bloodstream infections rose from 24% to 32%. Multivariable analysis identified device-specific rather than generic associations: mechanical ventilation with CRAB, urinary catheterization with CRE and inversely with CRAB, and endotracheal intubation with VRE. The case for pathogen-tailored rather than uniform prevention bundles follows directly. The authors are appropriately cautious that pandemic-era changes in sampling practice and case-mix may themselves have shaped the observed trends, a caveat that returns us to the denominators raised by Huang et al.

## Extending susceptibility testing beyond the culture laboratory

Wei et al. report a prospective multicenter evaluation of a stool-based multiplex RT-PCR assay detecting *23S rRNA* and *gyrA* mutations in *Helicobacter pylori* in 1,176 participants across four Chinese centers. Overall agreement with E-test phenotypes was 90.4% for clarithromycin and 89.1% for fluoroquinolones, rising to 95.9% and 94.7% against Sanger sequencing; concordance varied with MIC, with the best agreement at intermediate values. The significance lies less in the numbers than in the sample type. Decoupling resistance-guided eradication therapy from endoscopy and from the demanding microaerophilic culture of *H. pylori* makes the Maastricht VI recommendation for resistance-guided therapy operationally realistic in settings where neither is routinely available.

## Speed, breadth, and the limits of genotype

Li et al. compare targeted next-generation sequencing (tNGS) with culture and phenotypic susceptibility testing in 199 ICU patients with suspected ventilator-associated pneumonia. For pathogen identification, tNGS was markedly superior (sensitivity 98.98% versus 66.84% for culture) and roughly halved turnaround time (1.66 versus 3.00 days). For resistance, the picture is more instructive: overall genotype-phenotype consistency was 79.31%, excellent for *A. baumannii* carbapenem, penicillin, and cephamycin resistance (*bla*OXA-23 achieved 100% positive predictive value), but no fluoroquinolone or non-carbapenem beta-lactam determinant was recovered, and more than half of tetracycline resistance was missed. Discordance tracked with low pathogen abundance, and applying read-count thresholds for *APH*, *ANT*, *AAC(6’)*, and *sul* raised gene-level concordance from 68.15% to 75.93%. This candid accounting of where a genotypic method fails is more useful to the field than a headline sensitivity figure.

## Resolution: from isolate to mobile element

van Almsick et al. investigate seven patients with KPC-3-producing *K. pneumoniae* across three wards of a German tertiary center over six months using long-read whole genome sequencing. Core genome MLST resolved two unrelated clusters (ST307, n=5 and ST101, n=2). All isolates carried *bla*KPC-3 on IncF plasmids, but Mash distance-based comparison argued against plasmid transfer between clusters, while identifying a near-identical Tn4401a-like transposon shared by every isolate. Two lessons follow. First, the epidemiological unit of resistance spread is frequently smaller than the isolate and smaller than the plasmid, and surveillance that stops at species and susceptibility phenotype cannot see it. Second, a well-supported negative finding has operational value: excluding plasmid transmission redirected infection control toward the clonal and environmental routes that contact tracing had suggested, and the resulting measures contained the event.

## Complementarity, not substitution

Read together, these contributions describe complementary gains. Molecular and sequencing methods deliver speed (Li et al., 2025), reach beyond invasive sampling and culture (Wei et al.), and resolution down to the mobile element (van Almsick et al.). None displaces the isolate. Phenotypic susceptibility testing remains the arbiter where genotype and clinical course diverge, MICs remain necessary for dose optimization and breakpoint-based reporting, and genomic surveillance itself depends on a laboratory able to recover, identify, and bank organisms. The strategic conclusion for resource-constrained settings is therefore not to leapfrog culture but to secure it, and, as Huang et al. demonstrate, even well-equipped tertiary centers have considerable ground to make up on the prior question of whether anything is sent to the laboratory before the first dose.

We close with an observation we consider a finding rather than an omission. This Research Topic was framed around the paucity of diagnostic and surveillance data from LMICs; the manuscripts we were able to accept originate from China and Germany. The same inequity in laboratory infrastructure, research funding, and publication support that motivated the call also determines who is positioned to generate and disseminate these data. Closing that gap will require sustained investment in quality-assured routine bacteriology, regional sequencing capacity, and equitable research partnerships, alongside the technical advances documented here.

Priorities we would highlight for future work include reporting pre-treatment pathogen testing rates as a standard stewardship quality indicator, with explicit denominators and with cultures reported separately from inflammatory biomarkers; harmonized standards for genotype-based resistance reporting that state thresholds and known blind spots; embedding long-read sequencing with plasmid and mobile genetic element analysis in routine molecular surveillance; and implementation and cost-effectiveness evaluations of these tools in low-resource laboratories, so that improved diagnostics translate into local antibiograms, better empirical therapy and, ultimately, better patient outcomes.
